# Use of Glycolysis‐Enhancing Drugs and Risk of Parkinson's Disease

**DOI:** 10.1002/mds.29184

**Published:** 2022-08-22

**Authors:** Jacob E. Simmering, Michael J. Welsh, Jordan Schultz, Nandakumar S. Narayanan

**Affiliations:** ^1^ Department of Internal Medicine, Roy J. and Lucille A. Carver College of Medicine University of Iowa Iowa City Iowa USA; ^2^ Pappajohn Biomedical Institute, Roy J. and Lucille A. Carver College of Medicine University of Iowa Iowa City Iowa USA; ^3^ Department of Molecular Physiology and Biophysics, Roy J. and Lucille A. Carver College of Medicine University of Iowa Iowa City Iowa USA; ^4^ Department of Neurology, Roy J. and Lucille A. Carver College of Medicine University of Iowa Iowa City Iowa USA; ^5^ Howard Hughes Medical Institute, University of Iowa Iowa City Iowa USA; ^6^ Department of Psychiatry, Roy J. and Lucille A. Carver College of Medicine University of Iowa Iowa City Iowa USA; ^7^ Division of Pharmacy Practice and Sciences, College of Pharmacy University of Iowa Iowa City Iowa USA

**Keywords:** Parkinson's disease, epidemiology, pharmacoepidemiology

## Abstract

**Background:**

Terazosin (TZ) and closely related α1‐adrenergic receptor antagonists (doxazosin [DZ] and alfuzosin [AZ]) enhance glycolysis and reduce neurodegeneration in animal models. Observational evidence in humans from several databases supports this finding; however, a recent study has suggested that tamsulosin, the comparator medication, increases the risk of Parkinson's disease.

**Aims:**

We consider a different comparison group of men taking 5α‐reductase inhibitors (5ARIs) as a new, independent comparison allowing us to both obtain new estimates of the association between TZ/DZ/AZ and Parkinson's disease outcomes and validate tamsulosin as an active comparator.

**Methods:**

Using the Truven Health Analytics Marketscan database, we identified men without Parkinson's disease, newly started on TZ/DZ/AZ, tamsulosin, or 5ARIs. We followed these matched cohorts to compare the hazard of developing Parkinson's disease. We conducted sensitivity analyses using variable duration of lead‐in to mitigate biases introduced by prodromal disease.

**Results:**

We found that men taking TZ/DZ/AZ had a lower hazard of Parkinson's disease than men taking tamsulosin (hazard ratio (HR) = 0.71, 95% CI [confidence interval]: 0.65–0.77, n = 239,888) and lower than men taking 5ARIs (HR = 0.84, 95% CI: 0.75–0.94, n = 129,116). We found the TZ/DZ/AZ versus tamsulosin HR to be essentially unchanged with up to 5 years of lead‐in time; however, the TZ/DZ/AZ versus 5ARI effect became attenuated with longer lead‐in durations.

**Conclusions:**

These data suggest that men using TZ/DZ/AZ have a somewhat lower risk of developing Parkinson's disease than those using tamsulosin and a slightly lower risk than those using 5ARIs. © 2022 The Authors. *Movement Disorders* published by Wiley Periodicals LLC on behalf of International Parkinson and Movement Disorder Society

## Graphical abstract

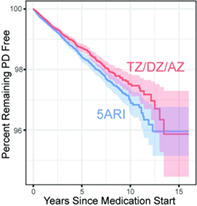

Parkinson's disease (PD) is a neurodegenerative disease with increasing overall prevalence as the population ages.^1^, ^2^ Although the causes of sporadic PD remain uncertain, there is substantial evidence implicating altered energy metabolism. Diverse genetic causes of PD, including *PINK1, parkin, DJ‐1, CHCHD2, α‐synuclein*, and *LRRK2* mutations,^3^, ^4^ impair energy metabolism, and the mitochondrial toxins 1‐methyl‐4‐phenyl‐1,2,3,6‐tetrahydropyridine and rotenone induce PD.^2^ Aging, the primary risk factor of PD, is associated with reduced brain energy metabolism, lower rates of mitochondrial biogenesis, and lower brain adenosine triphosphate (ATP) levels.^2^, ^5^, ^6^, ^7^ These diverse observations converge on impaired brain energy metabolism as a common pathogenic mechanism of PD. Consequently, enhancing energy metabolism might slow down or prevent PD.

Indeed, recent data suggest that enhancing glycolysis slows neurodegeneration. In particular, terazosin and the related α1‐adrenergic receptor antagonists doxazosin and alfuzosin (collectively TZ/DZ/AZ) bind to and activate phosphoglycerate kinase 1 (PGK1), the first ATP‐producing enzyme in glycolysis. In genetic and toxin‐induced models of PD in mice, rats, flies, and induced pluripotent stem cells from PD patients, TZ/DZ/AZ increased brain and cellular ATP levels and prevented or slowed neuron loss.^8^ TZ also increased brain ATP in a small pilot study in humans.^9^ In clinical practice, TZ/DZ/AZ are routinely used to treat benign prostatic hyperplasia (BPH), a common condition in older men. Tamsulosin, another α1‐adrenergic receptor antagonist, has similar clinical indications and effectiveness^10^ but does not engage PGK1.^8^ In addition, analysis of the Parkinson's Progression Markers Initiative found no difference in the change in motor performance between men treated with tamsulosin and untreated men, supporting a belief of an overall null effect of tamsulosin on PD pathogenesis.^8^ This creates natural comparison groups of men with similar health status who elect to undergo pharmaceutical treatment for BPH. Observational studies found that compared to tamsulosin, TZ/DZ/AZ use was associated with slower progression of motor impairment and complications in men with PD^8^ and lower incidence of PD in men across four databases in the United States,^11^, ^12^ Canada,^13^ and Denmark^11^ in analyses conducted by three independent research groups.

Recently, a report from Sasane and colleagues suggested that the decreased PD risk for people using TZ/DZ/AZ versus tamsulosin was due to tamsulosin increasing the risk of developing PD.^12^ In particular, in a comparison between men taking TZ/DZ/AZ and an untreated propensity‐score‐matched cohort, they found no difference in PD incidence, whereas a similar comparison between men taking tamsulosin and an untreated cohort found an increased PD incidence in tamsulosin users.^12^ These data were interpreted to suggest that tamsulosin increases the risk of PD, and the apparent reduction in risk for men taking TZ/DZ/AZ is the result of an improperly selected comparison group.

However, such a comparison has a high risk of reverse causality. Prodromal PD can cause urinary dysfunction,^14^ which is also a primary indication for treatment with TZ/DZ/AZ or tamsulosin. Any analysis^15^ that fails to consider the unobserved differences could find a spurious relationship between medication use and PD risk, especially in comparisons against men who are untreated or using non‐BPH medications. Therefore, it is common to restrict the sample to only people who meet the indication for treatment, often by making comparisons against those treated with a rival drug,^16^ and to delay the start of follow‐up to mitigate bias from prodromal or undiagnosed PD at baseline. These data‐centric, as opposed to model‐centric, controls reduce the unobserved confounding present in the data by decreasing the differences between treated and control participants through selection effects.

Consequently, we investigated PD risk with a new comparator group: men using the 5α‐reductase inhibitors (5ARIs) finasteride or dutasteride. Like α1‐adrenergic receptor antagonists, 5ARIs are commonly prescribed for BPH.^10^ We hypothesized that men taking TZ/DZ/AZ will have reduced hazard of developing PD compared to men using a 5ARI and that the hazard for men taking a 5ARI or tamsulosin will be similar. This comparison creates a new, independent test of both the effect of TZ/DZ/AZ and the suitability of tamsulosin as a comparator group. We tested these hypotheses using a propensity‐score‐matched cohort of men taking TZ/DZ/AZ, 5ARI, and tamsulosin in a large database of commercial health insurance claims.

## Patients and Methods

### Data Source

Health insurance claims for men newly started on TZ, DZ, AZ, tamsulosin, finasteride (5ARI), or dutasteride (5ARI) were obtained from the Truven Analytics Marketscan Commercial Claims and Encounters and Medicare Supplemental and Coordination of Benefits (MDCR) databases for 2001 to 2017. The Marketscan databases contain health insurance claims with diagnoses for all inpatient and outpatient encounters and, for enrollees with prescription drug coverage, claims with the National Drug Code (NDC) number of the dispensed medication. Secondary use of deidentified existing data, such as Truven Marketscan, is not considered human subjects research and is exempt from Institutional Review Board review.

### Cohort Construction

We identified all prescription events for one of the six medications of interest using the NDC numbers provided for each medication in the 2015 Redbook. For each enrollee, we then identified the first‐observed dispensing event. We required an enrollee to take only one of the three medication comparison groups and prohibited switching between groups. That is, an enrollee would only ever take TZ/DZ/AZ, tamsulosin, or 5ARI.

To ensure that the observed dispensing event reflected a newly started therapy, we required at least 365 days of continuous enrollment with prescription drug coverage before the index date. We required at least one additional dispensing claim in the year after the first dispensing to ensure we identified men who are likely users of the medication in practice and who do not experience immediate adverse events, such as orthostatic hypotension. As these drugs are rarely used in women, we required that enrollees be men. Both BPH, the most common indication for treatment, and PD are rare diagnoses below age 40 years, so we required enrollees to be aged at least 40 years at the index date. We required all enrollees to have at least 1 day of enrollment after the index date and to have been free of PD (see following case definition) on the index date. All enrollees were followed for up to 10 years. Men who left the Truven database without developing PD were censored at exit.

### PD Case Definition

We defined a person as having PD if he or she ever, in any setting, received the diagnosis of PD (ICD‐9‐CM: 332.0 or ICD‐10‐CM: G20) or had a prescription drug claim for levodopa (l‐dopa). The PD event date was taken as the first appearance of either the diagnosis or prescription dispensing event.

### Propensity‐Score Matching

Expecting differences between our cohorts, we used propensity‐score matching to reduce the imbalance. We estimated a propensity score, including year of commencing medication, age, incidence rate of outpatient visits during lookback, mean number of unique diagnoses recorded per outpatient visit during lookback, incidence rate of diagnoses made in outpatient settings during lookback, incidence rate of inpatient visits during lookback, history of BPH (ICD‐9‐CM: 600.xx or ICD‐10‐CM: N40.x), whether prostate‐specific antigen (PSA) levels were measured (CPT: 84152, 84153, 84154), if PSA levels were abnormal (ICD‐9‐CM: 790.93; ICD‐10‐CM: R97.2, R97.20, R97.21), whether a diagnosis of slow urinary stream was performed (ICD‐9‐CM: 788.62; ICD‐10‐CM: R39.12), whether a uroflow study was performed (ICD‐9‐CM procedure code: 89.24; ICD‐10‐CM procedure code: 4A1D75Z, CPT: 51736, 51741), whether a cystometrogram was collected (ICD‐9‐CM procedure code: 89.22; ICD‐10‐CM procedure code: 4A0D7BZ, 4A0D8BZ, 4A1D7BZ, 4A1D8BZ; CPT: 51725, 51726), whether a diagnosis of orthostatic hypotension was performed (ICD‐9‐CM: 458.0 or ICD‐10‐CM: I95.1), a diagnosis of other hypotension (ICD‐9‐CM: 458.1, 458.2x, 458.8, 458.9 or ICD‐10‐CM: I95.0, I95.2, I95.3), and the 30 Elixhauser comorbidity flags, as revised by AHRQ, using the R package icd.^17^, ^18^

For year of medication start, we included a series of dummy variables as we expected both changes in medication use patterns and rate of diagnosis of PD across our sample. In addition, insurance companies enter and leave the Truven project creating discontinuities in the cohorts. Including dummy variables for year accounts for these problems. We used splines of all the continuous variables to allow for nonlinear responses and estimated the propensity score using generalized additive models with a logistic link. Model estimation was performed using the R package mgcv^19^, ^20^ in R 4.0.4.^21^

We used a two‐step matching algorithm. First, we required the time from the medication start date to the end of enrollment to be the same (±90 days) to ensure balance in time‐at‐risk between the patients and controls. Second, within the set of possible matches with similar follow‐up, we used greedy nearest‐neighbor matching based on the estimated log odds.^22^ To ensure matches were high quality, we imposed a caliper equal to 20% of the pooled standard deviation of the log odds. We matched 1:1 without replacement. In the event of multiple equally good matches, we selected the matching control observation at random. We then had three matched cohorts for the three comparisons of interest: TZ/DZ/AZ versus tamsulosin, TZ/DZ/AZ versus 5ARI, and tamsulosin versus 5ARI.

### Assessing Propensity‐Score Match Balance

We compared the groups before and after matching on the variables included in the propensity‐score model. Our primary measure of balance was Cohen's d, a common measure of effect size. We defined the absolute value of Cohen's d of less than 0.1 as indicating minimal differences between the groups on a covariate.^23^, ^24^

### Survival Analysis

We calculate the time from medication start to PD diagnosis using the Kaplan–Meier estimator, visualizing both the curve and the days until 0.5%, 1%, 1.5%, and 2% cumulative incidence. Unfortunately, as less than 50% of the cohort develops PD, we are unable to estimate the median time until PD diagnosis. Using a simple mean instead of the Kaplan–Meier estimator would not incorporate the data from the censored cases. We tested whether the two survival curves were different using the nonparametric log‐rank test and quantified the difference using semiparametric Cox proportional hazards regression. For the Cox regression, we used robust standard errors clustered by the pair generated by propensity‐score matching. Deviation from the proportional hazards assumption was assessed using the Schoenfeld residuals. All survival analysis was performed using the R package survival.^25^

### Sensitivity Analyses

We assessed the robustness of our result using several sensitivity analyses. First and important, as PD has a long prodromal state, we conducted a series of sensitivity analyses starting follow‐up 1, 2, 3, 4, or 5 years after medication start. We also tested for violations of the proportional hazards assumption in these models. Second, we repeated our analysis using only the diagnosis of PD as a case‐defining event as l‐dopa may be used in other, although less common, conditions such as multiple system atrophy. Third, we repeated our analysis but required a diagnosis of BPH, slow urinary stream, or a procedure suggestive of urinary dysfunction to reduce unobserved heterogeneity through varying indications for treatment.

All data and code were checked by the Biostatistics, Epidemiology, and Research Design core as part of the Institute for Clinical and Translational Sciences at the University of Iowa. All code is available at https://github.com/iacobus42/parkinson-disease-5ari.

## Results

After propensity‐score matching, groups for all three comparisons were well balanced. All had an absolute value of Cohen's d < 0.10, our prespecified threshold for balance, with the majority being less than 0.01 (Tables S1–S3).

**FIG 1:**
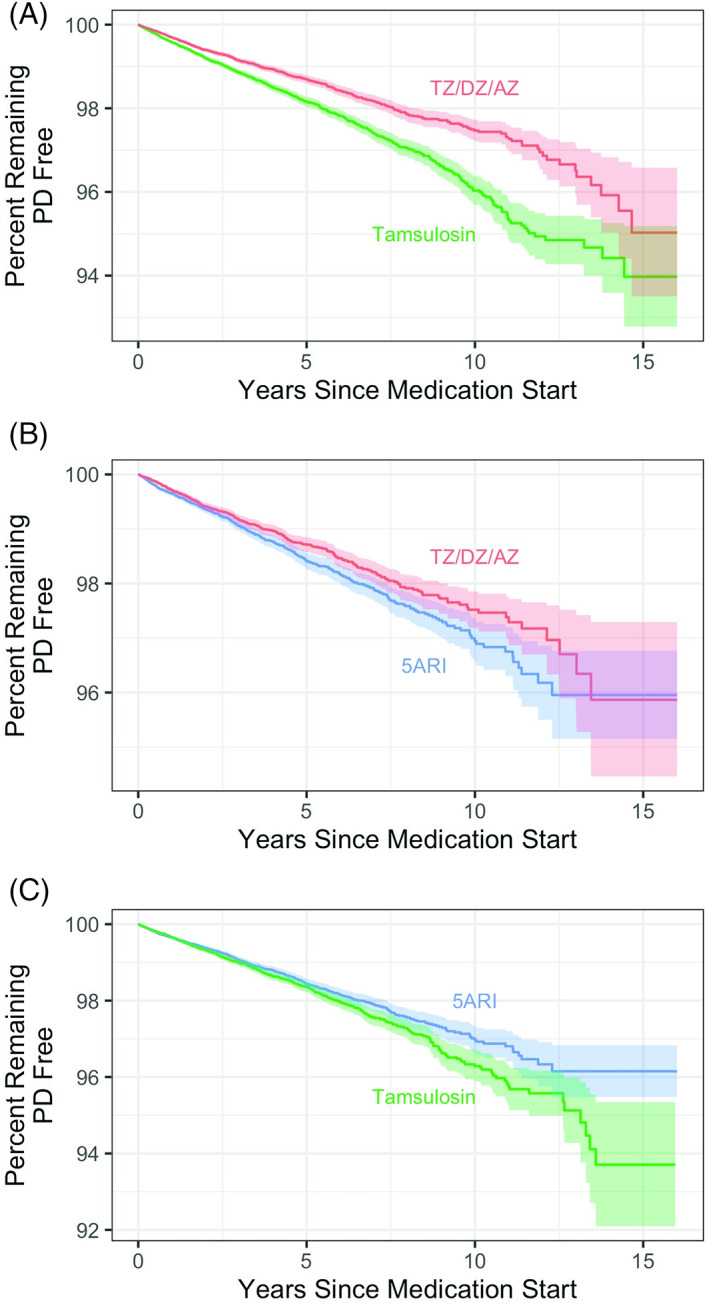
TZ/DZ/AZ (terazosin/doxazosin/alfuzosin) decreases the risk of PD compared to tamsulosin and 5ARI (5α‐reductase inhibitors). Kaplan–Meier survival curves for matched cohorts (**A**) for TZ/DZ/AZ versus tamsulosin, (**B**) for TZ/DZ/AZ versus 5ARI, and (**C**) for tamsulosin versus 5ARI.

Our matched cohort size and disease incidence are presented in Table 1. We observed 27.6 patients per 10,000 men per year taking TZ/DZ/AZ compared to 38.8 patients per 10,000 men taking tamsulosin (log‐rank *P*‐value <0.001). Similarly, men taking TZ/DZ/AZ had a lower incidence (26.5 per 10,000 per year) than matched men taking 5ARI (31.6 per 10,000 per year), a difference that was statistically significant (log‐rank *P*‐value = 0.003). The difference was smaller when comparing men taking tamsulosin, 35.0 patients per 10,000 men per year, to men taking 5ARI, 31.3 patients per 10,000 men per year (*P* = 0.024). Kaplan–Meier survival curves for all three comparisons are shown in Figure 1; see Table S4 for number at risk by year and Table S5 for days until 0.5%, 1.0%, 1.5%, and 2% incidence.

**TABLE 1:** Summary of matched cohort, number at risk, patients, incidence rates, and log‐rank test

Comparison	Medication	Number of enrollees	Person‐years of follow‐up time	Number of PD patients	Incidence of PD per 10,000 person‐years	Log‐rank test *P*‐value
TZ/DZ/AZ vs. tamsulosin	TZ/DZ/AZ	119,944	358,705	991	27.6	<2 × 10^−16^
	Tamsulosin	119,944	358,504	1391	38.8	
TZ/DZ/AZ vs. 5ARI	TZ/DZ/AZ	64,558	196,257	521	26.5	0.003
	5ARI	64,558	196,285	620	31.6	
Tamsulosin vs. 5ARI	Tamsulosin	78,747	241,542	846	35.0	0.024
	5ARI	78,747	241,923	757	31.3	

All groups were matched 1:1 based on a propensity score that included age, comorbidities, past medical utilization, and medication start date as well as approximate matching on the duration of postmedication start date enrollment time.

Abbreviations: PD, Parkinson's disease; TZ, terazosin; DZ, doxazosin; AZ, alfuzosin; 5ARI, 5α‐reductase inhibitors.

Semiparametric estimation with Cox proportional hazards regression provided similar results (Table 2). After matching based on propensity scores, we found a significant reduction in the hazard of PD for men taking TZ/DZ/AZ compared to tamsulosin (HR = 0.71, 95% CI [confidence interval]: 0.65–0.77). This effect was robust to changing the control group to men taking 5ARI, with a 16% reduction in hazard for men who took TZ/DZ/AZ (HR = 0.84, 95% CI: 0.75, 0.94). There was a significant difference in the hazard of PD for men using tamsulosin compared to men taking 5ARI (HR = 1.12, 95% CI: 1.01–1.23).

**TABLE 2:** Results of Cox proportional hazards regression

Comparison	Unadjusted			Propensity‐score matched			
	Hazard ratio	95% CI lower bound	95% CI upper bound	Hazard ratio	95% CI lower bound	95% CI upper bound	*P*‐value
TZ/DZ/AZ vs. tamsulosin	0.66	0.62	0.71	0.71	0.65	0.77	<0.001
TZ/DZ/AZ vs. 5ARI	0.88	0.80	0.97	0.84	0.75	0.94	0.003
Tamsulosin vs. 5ARI	1.33	1.23	1.44	1.12	1.01	1.23	0.024

Propensity‐score‐matched cohorts were matched 1:1 on a propensity score that included age, comorbidities, past medical utilization, and medication start date as well as approximate matching on the duration of postmedication start date enrollment time. Robust standard errors were clustered by pair on the matched data set. The second medication in each comparison is the reference group.

Abbreviations: CI, confidence interval; TZ, terazosin; DZ, doxazosin; AZ, alfuzosin; 5ARI, 5α‐reductase inhibitors.

### Sensitivity Analyses

Our first robustness check concerned the proportional hazards assumption of the Cox model. Schoenfeld residuals (Figure S1) showed nonsignificant correlation with time for the TZ/DZ/AZ versus tamsulosin (r = −0.029, *P* = 0.159) and TZ/DZ/AZ versus 5ARI (r = 0.003, *P* = 0.932) comparisons, whereas the tamsulosin versus 5ARI comparison did have significant correlation (r = 0.056, *P* = 0.026). We found relatively little change in the estimated hazard ratio when we include an interaction with a step function of time in Table S6 and Figure S2. The time‐dependent coefficients appear to be largely consistent with the time‐invariant estimates, although the time‐invariant TZ/DZ/AZ versus 5ARI hazard ratio may briefly overstate the effect during the second year of follow‐up.

Sensitivity analyses where we varied the start date of follow‐up by 1, 2, 3, 4, or 5 years found general consistency in the HR for TZ/DZ/AZ versus tamsulosin (HR varying from 0.71 to 0.68 with 0–5 years of lead‐in, all statistically significant) but attenuation for TZ/DZ/AZ versus 5ARI (HR increasing from 0.85 with no lead‐in to a nonsignificant 0.98 at 5 years) (Table 3). Inspection of the Schoenfeld residuals shows no evidence of concerning departures from the proportional hazards assumption in the TZ/DZ/AZ versus tamsulosin or TZ/DZ/AZ versus 5ARI models (Figures S3 and S4).

**TABLE 3:** Effect of variable lead‐in time before start of follow‐up

Comparison	Duration of lead‐in	Sample size	Hazard ratio	95% lower bound	95% upper bound
TZ/DZ/AZ vs. tamsulosin	0	239,896	0.71	0.65	0.77
	1	173,696	0.70	0.63	0.77
	2	120,076	0.69	0.61	0.78
	3	87,460	0.68	0.59	0.79
	4	63,924	0.68	0.58	0.80
	5	46,114	0.67	0.55	0.82
TZ/DZ/AZ vs. 5ARI	0	129,132	0.85	0.76	0.95
	1	94,218	0.87	0.75	1.00
	2	66,974	0.86	0.72	1.01
	3	48,478	0.80	0.95	0.98
	4	35,376	0.89	0.71	1.12
	5	25,652	0.98	0.73	1.31
Tamsulosin vs. 5ARI	0	157,502	1.14	1.03	1.25
	1	116,738	1.23	1.10	1.39
	2	83,332	1.15	1.00	1.33
	3	60,550	1.22	1.04	1.44
	4	44,056	1.21	1.00	1.47
	5	31,862	1.48	1.16	1.87

We varied the duration of lead‐in time from 0 to 5 years to mitigate the effects of prodromal or undetected PD at baseline. We found across a broad range of lead‐in windows little effect of the point estimate of the hazard ratio. The 95% confidence interval widened as a result of the decreasing sample size.

Abbreviations: TZ, terazosin; DZ, doxazosin; AZ, alfuzosin; 5ARI, 5α‐reductase inhibitors.

Additional sensitivity analyses suggested our results are generally consistent with the main result. First, considering only a diagnosis of PD as the exclusive outcome definition does not alter the results, with estimated HR for TZ/DZ/AZ versus tamsulosin 0.67 (95% CI: 0.61, 0.73), TZ/DZ/AZ versus 5ARI 0.81 (95% CI: 0.71, 0.92), and tamsulosin versus 5ARI 1.16 (95% CI: 1.04, 1.28). Second, requiring a diagnosis of BPH or urinary dysfunction to reduce unobserved heterogeneity also yielded directly consistent results, with estimated HR for TZ/DZ/AZ versus tamsulosin 0.84 (95% CI: 0.73, 0.96; n = 88,046), estimated HR for TZ/DZ/AZ versus 5ARI 0.82 (95% CI: 0.70, 0.97; n = 56,570), and the estimated HR for tamsulosin versus 5ARI reduced to null (HR = 0.99; 95% CI: 0.87, 1.14).

## Discussion

This study expands existing observational research, suggesting a possible neuroprotective role of TZ/DZ/AZ by directionally replicating the finding with a new comparison cohort of men taking a 5ARI. Our finding is convergent with the initial^11^ and replicated results,^12^, ^13^ finding lower hazard among men taking TZ/DZ/AZ against tamsulosin, and is robust to violations of the proportional hazards assumption. Including variable lead‐in periods of up to 5 years to mitigate potential biases due to prodromal disease showed consistent results for TZ/DZ/AZ versus tamsulosin, with only small changes in the estimate; however, the same models for TZ/DZ/AZ versus 5ARI showed evidence of attenuation to null effect with 5 years of lead‐in.

A key challenge in observational research, and pharmacoepidemiology in particular, is understanding the process that leads to treatment decisions. This is a particular problem when these factors are unobserved, a problem unaddressable by statistical adjustment or propensity‐score matching. Comparisons between men taking TZ/DZ/AZ, tamsulosin, or 5ARI are examples of the active comparator design. By conditioning on treatment and, indirectly, indication, we likely reduce some of the residual confounding relative to designs using untreated men as a comparison group.^16^ Indeed, in the sensitivity analysis requiring a diagnosis of BPH or urinary dysfunction in the lookback period, which reduces unobserved heterogeneity in the sample at the cost of a smaller sample size, we found directionally consistent and similar estimated effects for TZ/DZ/AZ versus both tamsulosin and 5ARI, whereas the tamsulosin versus 5ARI effect was reduced to nearly zero (HR = 0.99). The main results and the results observed in the sensitivity analyses support a finding of lower PD incidence among TZ/DZ/AZ users and either no effect or slightly increased hazard among tamsulosin users to plausible counterfactuals.

Although these changes in estimates seen with the requirement of a BPH diagnosis compared to the main results are intriguing as they converge with the expected null effect for tamsulosin versus 5ARI and found very similar effects for TZ/DZ/AZ for both the comparator groups, the divergence of the estimates obtained without the BPH restriction using propensity‐score matching suggests an important limitation and threat. Propensity‐score matching can balance only on observed variables. Although in this circumstance our results are qualitatively consistent, such a large change in the estimates with restriction compared to the entire data set with propensity‐score matching suggests at least some variables related to treatment behavior and PD risk are not balanced by our matching scheme. Foremost would be concerns about unmeasured indications (lower urinary tract symptom burden) or contraindications (especially those related to orthostatic hypotension, as tamsulosin is likely preferred in such situations) that are related to preclinical and the earliest manifestations of PD.

Our study has some limitations. The relationship between prodromal PD and lower urinary tract symptoms may introduce bias in our comparisons. Our analyses using variable lead‐in periods of up to 5 years to reduce or eliminate this threat suggest that prodromal disease is not a likely threat to the TZ/DZ/AZ versus tamsulosin results but that the TZ/DZ/AZ versus 5ARI estimate may attenuate to null effect during longer lead‐in periods. The strength of the 5ARI comparison is therefore necessarily weaker and should be the subject of future investigation.

In addition, administrative data are limited in that they were not originally collected for research requirements. The sensitivity of diagnosis and procedure codes to detect a condition in administrative data, compared to chart review, may be low. This is a particular limitation in that mild BPH, hypertension, or orthostatic hypotension may not be recorded as a diagnosis but may influence treatment selection. Of these, underreporting of orthostatic hypotension is the greatest threat to our results as it is both a symptom of prodromal PD and a factor that influences prescribing away from TZ/DZ/AZ in a mirror image of the issue with urinary symptoms. Although we include a diagnosis of orthostatic hypotension in our propensity scores, if it is underreported, we may have imbalance between the groups. Other variables related to PD risk, such as smoking status or race, are either poorly captured or not reported at all in the Truven Health Analytics database. These factors, together with the relatively short duration of follow‐up in our data and the duration of medication use, present challenges to a causal interpretation of the results and highlight the need for a trial to test and describe any causal effects.

The results of our study, together with four previous retrospective studies from four independent data sets in three countries, provide remarkably consistent evidence that the use of TZ/DZ/AZ is associated with a decreased risk of developing PD. These results, combined with observations that impaired brain energy metabolism may predispose to PD, with the mechanistic discovery that TZ enhances glycolysis and energy metabolism, and with findings that TZ slows down or prevents neurodegeneration in multiple toxin‐induced and genetic models of PD,^8^ provide convergent evidence that TZ/DZ/AZ slows down neurodegeneration in PD. Ultimately, additional epidemiological investigation and a successful randomized controlled trial of TZ/DZ/AZ as a disease‐modifying therapy are needed before starting the randomized controlled trial ultimately needed to show a causal effect of TZ/DZ/AZ on the risk of PD.

## Author Roles

J.E.S. designed and conducted the analysis, provided an initial draft of the manuscript, and was involved in critical revision and preparation of the manuscript.

M.J.W. contributed to critical appraisal of the study design and critical revision and preparation of the manuscript.

J.S. contributed to critical revision and preparation of the manuscript.

N.S.N. contributed to critical appraisal of the study design and critical revision and preparation of the manuscript.

## Full financial disclosures for the previous 12 months

J.E.S. received the KL2 Career Development Award from the University of Iowa Institute for Clinical and Translational Science and Environmental Sciences Career Development Award from the University of Iowa Environmental Health Science Research Center and receives funding from the NIH. He has previously been supported by the Iowa Neuroscience Institute as a faculty fellow.

M.J.W. is a principal investigator on a T32 multidisciplinary lung research career development program from the NIH, a project leader on two NIH P01 cystic fibrosis grants, and a Howard Hughes Medical Institute investigator. He is an investigator on a project developing novel agents to enhance energy metabolism for Alzheimer's disease from the Thome Foundation and a coinvestigator on a project assessing target engagement for TZ in Parkinson's disease funded by The Michael J. Fox Foundation.

J.S. received a career development award from NINDS and The Michael J. Fox Foundation.

N.S.N. receives support from the NIH, DOD, and the University of Iowa.

## Author Contributions

Conception and design (JES, MJW, JLS, NSN), data analysis (JES), drafting and revising text (JES, MJW, JLS, NSN).

## Supporting information


**APPENDIX S1.** Supporting Information

## Data Availability

Truven Health Analytics Marketscan databases are used under license from Truven Health Analytics.
