# Supplementary material for: Use of Glycolysis‐Enhancing Drugs and Risk of Parkinson's Disease
Source: Mov Disord. 2022 Aug 22;37(11):2210–6. doi: 10.1002/mds.29184 (PMC9669185; doi:10.1002/mds.29184)

Use of glycolysis-enhancing drugs is associated with a reduced risk of Parkinson's disease: Supplemental

Contents

[Table S1: Summary Measures of TZ/DZ/AZ Versus Tamsulosin Cohort. 2](#_Toc105770977)

[Table S2: Summary Measures of TZ/DZ/AZ Versus 5ARI Cohort. 4](#_Toc105770978)

[Table S3: Summary Measures of Tamsulosin Versus 5ARI Cohort 6](#_Toc105770979)

[Table S4: Number Remaining Enrolled and PD-Free by Years of Follow-Up. 8](#_Toc105770980)

[Table S5: Mean Number of Days Until 0.5, 1.0, 1.5, or 2.0% Incidence. 8](#_Toc105770981)

[Table S6: Cox Regression with Time Dependent Coefficients 9](#_Toc105770982)

[Figure S1: Schoenfeld Residuals for the Matched Cohort. 9](#_Toc105770983)

[Figure S2: Time Dependent Effects, Schoenfeld Residuals, and Overall HR. 10](#_Toc105770984)

[Figure S3: Time-Dependent Log Hazard Ratios by Lead-In Duration. 11](#_Toc105770985)

[Figure S4: Schoenfeld Residuals at Different Lead-In Windows. 12](#_Toc105770986)

Table S1: Summary Measures of TZ/DZ/AZ Versus Tamsulosin Cohort. Dichotomous variables are reported as precents and continuous variables are reported as the median and inner quartile range. Cohen’s d is included as a measure of difference between the two groups. Rows with absolute values of d greater than 0.1, our threshold for balance, are bolded.

|  | **Before Matching** | | | **After Matching** | | |
| --- | --- | --- | --- | --- | --- | --- |
| **Variable** | **TZ/DZ/AZ** | **Tamsulosin** | **d** | **TZ/DZ/AZ** | **Tamsulosin** | **d** |
| N | 124,905 | 429,741 |  | 119,944 | 119,944 |  |
| Duration of… |  |  |  |  |  |  |
| Lookback in Years | 2.9  (1.7, 5.2) | 3.6  (3.0, 6.3) | -0.24 |  |  |  |
| Follow-up in Years | 1.96  (0.93, 4.19) | 1.90  (0.86, 3.78) | 0.09 |  |  |  |
| Age | 62  (55, 70) | 62  (56, 71) | -0.05 | 62  (55, 70) | 62  (55, 70) | -0.02 |
| Annual Rate of Inpatient | 0.00  (0.00, 0.12) | 0.00  (0.00, 0.21) | -0.08 | 0.00  (0.00, 0.13) | 0.00  (0.00, 0.14) | -0.01 |
| Annual Rate of Outpatient | 9  (5, 16) | 10  (5, 18) | -0.07 | 9  (5, 16) | 9  (5, 16) | -0.01 |
| Mean Number of Diagnoses | **1.37**  **(1.18, 1.83)** | **1.56**  **(1.27, 2.09)** | **-0.28** | 1.38  (1.19, 1.86) | 1.39  (1.19, 1.87) | -0.01 |
| Annual Rate of Outpatient Diagnoses | **14**  **(7, 25)** | **16**  **(9, 29)** | **-0.13** | 14  (7, 25) | 14  (7, 25) | -0.01 |
| Year of Medication Start | **2009**  **(2006, 2012)** | **2012**  **(2008, 2014)** | **-0.48** | 2009  (2006, 2012) | 2009  (2006, 2012) | -0.01 |
| Percent With BPH Diagnosis | **33.6** | **41.9** | **-0.17** | 34.3 | 34.9 | -0.01 |
| Percent With PSA Measurement | 57.3 | 57.5 | 0.00 | 57.2 | 57.7 | -0.01 |
| Percent With Abnormal PSA | **11.2** | **16.2** | **-0.14** | 11.5 | 11.7 | -0.01 |
| Percent With Slow Urinary Stream | 2.3 | 3.2 | -0.05 | 2.4 | 2.6 | -0.01 |
| Percent with Uroflow Study | 6.0 | 6.5 | -0.02 | 6.4 | 6.2 | -0.01 |
| Percent With Cystometrogram | 0.7 | 0.8 | -0.01 | 0.7 | 0.8 | 0.00 |
| Percent With Orthostatic Hypotension Diagnosis | 0.7 | 1.2 | -0.04 | 0.7 | 0.8 | -0.01 |
| Percent With Other Hypotension Diagnosis | 1.7 | 2.9 | -0.08 | 1.8 | 1.8 | 0.00 |
| Percent With Diagnosis of Anxiety | 6.2 | 8.9 | **-0.10** | 6.4 | 6.5 | 0.00 |
| Percent With Diagnosis of Erectile Dysfunction | 8.9 | 10.4 | -0.05 | 9.1 | 9.3 | -0.01 |
| Percent With Comorbidity… |  |  |  |  |  |  |
| Alcohol Abuse | 2.1 | 2.6 | -0.03 | 2.2 | 2.2 | -0.01 |
| Anemia | 13.4 | 15.5 | -0.06 | 13.5 | 13.6 | 0.00 |
| Blood Loss | 1.2 | 1.6 | -0.04 | 1.2 | 1.2 | 0.00 |
| Heart Failure | 8.0 | 9.6 | -0.06 | 8.1 | 8.3 | -0.01 |
| Coagulopathy | 2.9 | 4.3 | -0.07 | 2.9 | 3.0 | 0.00 |
| Depression | 7.0 | 9.8 | -0.10 | 7.2 | 7.3 | 0.00 |
| Diabetes (Uncomplicated) | 28.9 | 27.7 | 0.03 | 29.0 | 29.1 | 0.00 |
| Diabetes (Complicated) | 11.3 | 10.6 | 0.02 | 11.4 | 11.5 | 0.00 |
| Drug Abuse | 1.0 | 1.5 | -0.04 | 1.1 | 1.1 | 0.00 |
| Electrolyte Disorders | 11 | 13 | -0.05 | 11.0 | 11.1 | 0.00 |
| HIV | 0.4 | 0.3 | 0.01 | 0.4 | 0.4 | 0.00 |
| Hypertension (Uncomplicated) | **69.4** | **62.0** | **0.15** | 69.2 | 69.2 | -0.01 |
| Hypertension (Complicated) | **16.4** | **12.9** | **0.10** | 16.2 | 16.6 | -0.01 |
| Hypothyroidism | 7.7 | 9.9 | -0.07 | 7.9 | 8.0 | -0.01 |
| Liver Disease | 3.8 | 5.6 | -0.08 | 3.9 | 4.0 | 0.00 |
| Lymphoma | 1.0 | 1.4 | -0.04 | 1.1 | 1.1 | 0.00 |
| Metastatic Cancer | 1.2 | 2.5 | -0.08 | 1.3 | 1.3 | 0.00 |
| Other Neuro. Conditions | 5.8 | 9.2 | -0.12 | 6.0 | 6.1 | -0.01 |
| Obesity | 9.1 | 11 | -0.06 | 9.4 | 9.4 | 0.00 |
| Paralysis | 1.6 | 2.4 | -0.05 | 1.7 | 1.7 | 0.00 |
| Pulmonary Hypertension | 1.9 | 3.0 | -0.07 | 2.0 | 2.1 | -0.01 |
| Psychoses | 5.4 | 7.2 | -0.07 | 5.5 | 5.7 | -0.01 |
| Peptic Ulcer Disease | 0.2 | 0.4 | -0.03 | 0.2 | 0.2 | 0.00 |
| COPD | **19.0** | **23.8** | **-0.12** | 19.4 | 19.6 | -0.01 |
| Peripheral Vascular Disease | 12.0 | 14.6 | -0.08 | 12.2 | 12.4 | -0.01 |
| Renal Failure | 9.5 | 7.1 | 0.09 | 9.4 | 9.5 | 0.00 |
| Rheumatoid Arthritis | 3.7 | 5.0 | -0.06 | 3.8 | 3.8 | 0.00 |
| Solid Tumor | **10.9** | **16.8** | **-0.16** | 11.3 | 11.4 | 0.00 |
| Valvular Disease | 11.1 | 14.1 | -0.09 | 11.3 | 11.5 | -0.01 |
| Weight Loss | 2.7 | 4.5 | -0.09 | 2.7 | 2.8 | 0.00 |

Table S2: Summary Measures of TZ/DZ/AZ Versus 5ARI Cohort. Dichotomous variables are reported as precents and continuous variables are reported as the median and inner quartile range. Cohen’s d is included as a measure of difference between the two groups. Rows with absolute values of d greater than 0.1, our threshold for balance, are bolded.

|  | **Before Matching** | | | **After Matching** | | |
| --- | --- | --- | --- | --- | --- | --- |
| **Variable** | **TZ/DZ/AZ** | **5ARI** | **d** | **TZ/DZ/AZ** | **5ARI** | **d** |
| N | 124,905 | 79,133 |  | 64,558 | 64,558 |  |
| Duration of… |  |  |  |  |  |  |
| Lookback in Years | 2.85  (1.71, 5.18) | 3.15  (1.83, 5.57) | -0.07 |  |  |  |
| Follow-up in Years | 1.96  (0.93, 4.19) | 2.23  (0.98, 4.43) | -0.05 |  |  |  |
| Age | 62  (55, 70) | 62  (54, 70) | 0.05 | 61  (54, 70) | 61  (54, 70) | -0.01 |
| Annual Rate of Inpatient | **0.00**  **(0.00, 0.12)** | **0.00**  **(0.00, 0.00)** | **0.11** | 0.00  (0.00, 0.00) | 0.00  (0.00, 0.00) | 0.00 |
| Annual Rate of Outpatient | 9  (5, 16) | 9  (5, 16) | 0.05 | 9  (5, 16) | 9  (5, 16) | 0.00 |
| Mean Number of Diagnoses | 1.37  (1.18, 1.83) | 1.40  (1.20, 1.87) | -0.04 | 1.39  (1.19, 1.86) | 1.40  (1.19, 1.88) | -0.02 |
| Annual Rate of Outpatient Diagnoses | 14  (7, 25) | 13  (7, 24) | 0.04 | 13  (7, 24) | 13  (7, 24) | -0.01 |
| Year of Medication Start | **2009**  **(2006, 2012)** | **2010**  **(2008, 2013)** | **-0.22** | 2010  (2007, 2013) | 2010  (2007, 2012) | -0.03 |
| Percent With BPH Diagnosis | **33.6** | **45.0** | **-0.24** | 42.5 | 51.8 | 0.01 |
| Percent With PSA Measurement | 57.3 | 60.7 | -0.07 | 59.0 | 59.5 | -0.01 |
| Percent With Abnormal PSA | **11.2** | **29.7** | **0.49** | 19.9 | 20.6 | -0.02 |
| Percent With Slow Urinary Stream | 2.3 | 2.4 | -0.01 | 2.6 | 2.5 | 0.01 |
| Percent with Uroflow Study | 6.0 | 7.0 | -0.04 | 7.3 | 6.9 | 0.02 |
| Percent With Cystometrogram | 0.7 | 0.7 | 0.00 | 0.8 | 0.7 | 0.01 |
| Percent With Orthostatic Hypotension Diagnosis | 0.7 | 0.9 | -0.03 | 0.8 | 0.9 | 0.00 |
| Percent With Other Hypotension Diagnosis | 1.7 | 1.8 | 0.01 | 1.8 | 1.8 | 0.00 |
| Percent With Diagnosis of Anxiety | 6.2 | 6.8 | -0.03 | 6.8 | 6.8 | 0.00 |
| Percent With Diagnosis of Erectile Dysfunction | 8.9 | 9.0 | 0.00 | 9.2 | 9.1 | 0.00 |
| Percent With Comorbidity… |  |  |  |  |  |  |
| Alcohol Abuse | 2.1 | 1.4 | 0.05 | 1.6 | 1.6 | 0.00 |
| Anemia | 13.4 | 11.4 | 0.06 | 11.6 | 11.7 | 0.00 |
| Blood Loss | 1.2 | 1.1 | 0.01 | 1.1 | 1.1 | 0.00 |
| Heart Failure | 8.0 | 6.6 | 0.05 | 6.7 | 6.8 | 0.00 |
| Coagulopathy | 2.9 | 2.9 | 0.00 | 2.9 | 2.9 | 0.00 |
| Depression | 7.0 | 6.8 | 0.01 | 7.1 | 7.0 | 0.00 |
| Diabetes (Uncomplicated) | **28.9** | **19.8** | **0.21** | 21.6 | 21.6 | 0.00 |
| Diabetes (Complicated) | **11.3** | **6.3** | **0.17** | 7.1 | 7.1 | 0.00 |
| Drug Abuse | 1.0 | 0.7 | 0.04 | 0.8 | 0.8 | 0.00 |
| Electrolyte Disorders | **10.8** | **7.3** | **0.12** | 7.9 | 7.9 | 0.00 |
| HIV | 0.4 | 0.7 | 0.04 | 0.6 | 0.6 | 0.00 |
| Hypertension (Uncomplicated) | **69.4** | **52.8** | **0.35** | 56.3 | 56.3 | 0.00 |
| Hypertension (Complicated) | **16.4** | **9.9** | **0.19** | 10.8 | 10.9 | 0.00 |
| Hypothyroidism | 7.7 | 8.8 | -0.04 | 8.4 | 8.5 | -0.01 |
| Liver Disease | 3.8 | 3.1 | 0.04 | 3.3 | 3.3 | 0.00 |
| Lymphoma | 1.0 | 1.0 | 0.00 | 1.1 | 1.0 | 0.00 |
| Metastatic Cancer | 1.2 | 1.4 | -0.01 | 1.4 | 1.4 | 0.00 |
| Other Neuro. Conditions | 5.8 | 5.6 | 0.01 | 5.7 | 5.7 | 0.00 |
| Obesity | 9.1 | 5.6 | 0.13 | 6.3 | 6.4 | 0.01 |
| Paralysis | 1.6 | 1.2 | 0.03 | 1.3 | 1.3 | 0.00 |
| Pulmonary Hypertension | 1.9 | 1.9 | 0.00 | 1.9 | 1.9 | 0.00 |
| Psychoses | 5.4 | 5.2 | 0.01 | 5.4 | 5.3 | 0.01 |
| Peptic Ulcer Disease | 0.2 | 0.2 | 0.00 | 0.2 | 0.2 | 0.00 |
| COPD | 19.0 | 17.7 | 0.03 | 18.1 | 18.0 | 0.00 |
| Peripheral Vascular Disease | 12.0 | 10.4 | 0.05 | 10.7 | 10.7 | 0.00 |
| Renal Failure | **9.5** | **4.3** | **0.20** | 4.8 | 5.0 | -0.01 |
| Rheumatoid Arthritis | 3.7 | 3.5 | 0.01 | 3.6 | 3.6 | 0.00 |
| Solid Tumor | 10.9 | 13.1 | -0.07 | 12.4 | 12.4 | 0.00 |
| Valvular Disease | 11.1 | 11.5 | -0.01 | 11.3 | 11.3 | 0.00 |
| Weight Loss | 2.7 | 2.8 | -0.01 | 2.8 | 2.8 | 0.00 |

Table S3: Summary Measures of Tamsulosin Versus 5ARI Cohort. Dichotomous variables are reported as precents and continuous variables are reported as the median and inner quartile range. Cohen’s d is included as a measure of difference between the two groups. Rows with absolute values of d greater than 0.1, our threshold for balance, are bolded.

|  | **Before Matching** | | | **After Matching** | | |
| --- | --- | --- | --- | --- | --- | --- |
| **Variable** | **Tamsulosin** | **5ARI** | **d** | **Tamsulosin** | **5ARI** | **d** |
| N | 429,741 | 79,133 |  | 78,747 | 78,747 |  |
| Duration of… |  |  |  |  |  |  |
| Lookback in Years | **3.6**  **(2.0, 6.3)** | **3.1**  **(1.8, 5.6)** | **0.18** |  |  |  |
| Follow-up in Years | **1.90**  **(0.86, 3.78)** | **2.23**  **(0.98, 4.43)** | **-0.14** |  |  |  |
| Age | **62**  **(56, 71)** | **62**  **(54, 70)** | **0.10** | 62  (54, 71) | 62  (54, 70) | 0.00 |
| Annual Rate of Inpatient | **0.00**  **(0.00, 0.21)** | **0.00**  **(0.00, 0.00)** | **0.18** | 0.00  (0.00, 0.00) | 0.00  (0.00, 0.00) | 0.02 |
| Annual Rate of Outpatient | **10**  **(5, 18)** | **9**  **(5, 16)** | **0.11** | 9  (5, 16) | 9  (5, 16) | 0.01 |
| Mean Number of Diagnoses | **1.56**  **(1.27, 2.09)** | **1.40**  **(1.20, 1.87)** | **0.24** | 1.41  (1.20, 1.88) | 1.41  (1.20, 1.87) | 0.01 |
| Annual Rate of Outpatient Diagnoses | **16**  **(9, 29)** | **13**  **(7, 24)** | **0.16** | 13  (7, 24) | 13  (7, 24) | 0.02 |
| Year of Medication Start | **2012**  **(2008, 2014)** | **2010**  **(2008, 2013)** | **0.27** | 2010  (2008, 2013) | 2010  (2008, 2013) | 0.01 |
| Percent With BPH Diagnosis | 41.9 | 45.0 | -0.06 | 45.0 | 44.9 | 0.00 |
| Percent With PSA Measurement | 57.5 | 60.7 | -0.07 | 60.7 | 60.6 | 0.00 |
| Percent With Abnormal PSA | **16.2** | **29.7** | **-0.35** | 29.7 | 29.5 | 0.00 |
| Percent With Slow Urinary Stream | 3.2 | 2.4 | 0.05 | 2.5 | 2.5 | 0.00 |
| Percent with Uroflow Study | 6.5 | 7.0 | -0.02 | 7.3 | 7.0 | 0.01 |
| Percent With Cystometrogram | 0.8 | 0.7 | 0.01 | 0.7 | 0.7 | 0.01 |
| Percent With Orthostatic Hypotension Diagnosis | 1.2 | 0.9 | 0.02 | 1.0 | 0.9 | 0.00 |
| Percent With Other Hypotension Diagnosis | 2.9 | 1.8 | 0.07 | 1.9 | 1.8 | 0.00 |
| Percent With Diagnosis of Anxiety | 8.9 | 6.8 | 0.08 | 7.0 | 6.8 | 0.01 |
| Percent With Diagnosis of Erectile Dysfunction | 10.4 | 9.0 | 0.05 | 9.1 | 9.0 | 0.00 |
| Percent With Comorbidity… |  |  |  |  |  |  |
| Alcohol Abuse | 2.6 | 1.4 | 0.08 | 1.5 | 1.4 | 0.01 |
| Anemia | 15.5 | 11.4 | 0.12 | 11.6 | 11.5 | 0.00 |
| Blood Loss | 1.6 | 1.1 | 0.04 | 6.8 | 6.6 | 0.01 |
| Heart Failure | 9.6 | 6.6 | 0.11 | 3.0 | 2.9 | 0.01 |
| Coagulopathy | 4.3 | 2.9 | 0.07 | 2.9 | 3.0 | 0.01 |
| Depression | 9.8 | 6.8 | 0.11 | 6.9 | 6.8 | 0.00 |
| Diabetes (Uncomplicated) | **27.7** | **19.8** | **0.18** | 20.4 | 19.9 | 0.01 |
| Diabetes (Complicated) | **10.6** | **6.3** | **0.14** | 6.7 | 6.4 | 0.02 |
| Drug Abuse | 1.5 | 0.7 | 0.07 | 0.8 | 0.7 | 0.02 |
| Electrolyte Disorders | **12.6** | **7.3** | **0.16** | 7.6 | 7.4 | 0.01 |
| HIV | 0.3 | 0.7 | -0.06 | 0.7 | 0.7 | 0.00 |
| Hypertension (Uncomplicated) | **62.0** | **52.8** | **0.19** | 53.0 | 52.9 | 0.00 |
| Hypertension (Complicated) | 12.9 | 9.9 | 0.09 | 10.2 | 9.9 | 0.01 |
| Hypothyroidism | 9.9 | 8.8 | 0.04 | 9.0 | 8.8 | 0.01 |
| Liver Disease | **5.6** | **3.1** | **0.11** | 3.3 | 3.1 | 0.01 |
| Lymphoma | 1.4 | 1.0 | 0.03 | 1.1 | 1.0 | 0.01 |
| Metastatic Cancer | 2.5 | 1.4 | 0.07 | 1.5 | 1.4 | 0.01 |
| Other Neuro. Conditions | **9.2** | **5.6** | **0.13** | 5.8 | 5.6 | 0.01 |
| Obesity | **11.0** | **5.6** | **0.18** | 6.0 | 5.7 | 0.01 |
| Paralysis | 2.4 | 1.2 | 0.08 | 1.3 | 1.2 | 0.01 |
| Pulmonary Hypertension | 3.0 | 1.9 | 0.07 | 2.1 | 1.9 | 0.01 |
| Psychoses | 7.2 | 5.2 | 0.08 | 5.3 | 5.2 | 0.01 |
| Peptic Ulcer Disease | 0.4 | 0.2 | 0.03 | 0.3 | 0.2 | 0.01 |
| COPD | **23.8** | **17.7** | **0.15** | 18.0 | 17.7 | 0.01 |
| Peripheral Vascular Disease | **14.6** | **10.4** | **0.12** | 10.8 | 10.5 | 0.01 |
| Renal Failure | **7.1** | **4.3** | **0.11** | 4.5 | 4.4 | 0.01 |
| Rheumatoid Arthritis | 5.0 | 3.5 | 0.07 | 3.7 | 3.5 | 0.01 |
| Solid Tumor | 16.8 | 13.1 | 0.10 | 14.0 | 13.2 | 0.02 |
| Valvular Disease | 14.1 | 11.5 | 0.08 | 11.8 | 11.5 | 0.01 |
| Weight Loss | 4.5 | 2.8 | 0.08 | 2.9 |  |  |

# Table S4: Number Remaining Enrolled and PD-Free by Years of Follow-Up.

| Years of Follow-Up | TZ/DZ/AZ vs Tamsulosin | | TZ/DZ/AZ vs 5ARI | | Tamsulosin vs 5ARI | |
| --- | --- | --- | --- | --- | --- | --- |
|  | TZ/DZ/AZ | Tamsulosin | TZ/DZ/AZ | 5ARI | Tamsulosin | 5ARI |
| 1 | 87,067 | 86,834 | 47,125 | 47,223 | 58,426 | 58,387 |
| 2 | 60,535 | 60,719 | 33,552 | 33,639 | 41,987 | 41,709 |
| 3 | 44,272 | 44,273 | 24,446 | 24,454 | 30,486 | 30,354 |
| 4 | 32,461 | 32,391 | 17,858 | 17,905 | 22,078 | 22,122 |
| 5 | 23,443 | 23,424 | 12,986 | 12,985 | 16,102 | 16,032 |
| 6 | 16,894 | 16,877 | 9,341 | 9,315 | 11,533 | 11,475 |
| 7 | 12,166 | 12,158 | 6,645 | 6,648 | 8,111 | 8,076 |
| 8 | 8,306 | 8,2930 | 4,493 | 4,504 | 5,463 | 5,471 |
| 9 | 5,535 | 5,529 | 2,975 | 2,993 | 3,542 | 3,584 |
| 10 | 3,484 | 3,479 | 1,877 | 1,865 | 2,204 | 2,238 |
| 11 | 2,124 | 2,122 | 1,072 | 1,089 | 1,301 | 1,316 |
| 12 | 1,133 | 1,121 | 511 | 536 | 658 | 643 |
| 13 | 629 | 646 | 270 | 266 | 327 | 328 |
| 14 | 304 | 317 | 127 | 115 | 161 | 163 |
| 15 | 121 | 117 | 35 | 34 | 48 | 43 |
| 16 | 2 | 3 | 2 | 2 | 1 | 2 |

Table S5: Mean Number of Days Until 0.5, 1.0, 1.5, or 2.0% Incidence. Values of reported as days until the cumulative incidence hits 0.5, 1.0, 1.5, or 2.0 percent and 95% CIs derived from the Kaplan Meier estimator.

|  | TZ/DZ/AZ vs Tamsulosin | | TZ/DZ/AZ vs 5ARI | | Tamsulosin vs 5ARI | |
| --- | --- | --- | --- | --- | --- | --- |
|  | TZ/DZ/AZ | Tamsulosin | TZ/DZ/AZ | 5ARI | Tamsulosin | 5ARI |
| 0.5% | 595 (547, 651) | 442 (412, 496) | 640 (596, 691) | 556 (495, 649) | 530 (482, 581) | 557 (467, 649) |
| 1.0% | 1,317 (1,237, 1,481) | 958 (890, 1,015) | 1,342 (1,188, 1,562) | 1,160 (1,064, 1,307) | 1,089 (980, 1,203) | 1,175 (1,067, 1,279) |
| 1.5% | 2,100 (1,968, 2,264) | 1,463 (1,380, 1,584) | 2,140 (1,995, 2,369) | 1,746 (1,161, 1,943) | 1,665 (1,519, 1,822) | 1,778 (1,666, 1,943) |
| 2.0% | 2,768 (2,578, 3,030) | 1,996 (1,891, 2,156) | 2,815 (2,57, 3,352) | 2,385 (2,183, 2,702) | 2,160 (1,995, 2,365) | 2,385 (2,183, 2,694) |

Table S6: Cox Regression with Time Dependent Coefficients. Data are the estimated time dependent hazard ratios and 95% CIs. As time increases, the sample size decreases considerably reducing precision of the estimates; however, the results are largely consistent with overall main model.

| Year | TZ/DZ/AZ vs Tamsulosin | TZ/DZ/AZ vs 5ARI | Tamsulosin vs 5ARI |
| --- | --- | --- | --- |
| 0-1 | 0.72 (0.62, 0.83) | 0.80 (0.65, 0.99) | 0.98 (0.82, 1.17) |
| 1-2 | 0.70 (0.67, 0.96) | 1.01 (0.78, 1.30) | 1.18 (0.95, 1.48) |
| 2-3 | 0.73 (0.58, 0.91) | 0.81 (0.59, 1.10) | 1.10 (0.85, 1.42) |
| 3-4 | 0.58 (0.44, 0.76) | 0.69 (0.47, 1.02) | 1.28 (0.94, 1.75) |
| 4-5 | 0.72 (0.52, 0.97) | 0.74 (0.50, 1.12) | 0.80 (0.56, 1.13) |
| 5-7.5 | 0.72 (0.56, 0.92) | 0.95 (0.66, 1.36) | 1.38 (1.03, 1.85) |
| 7.5-10 | 0.52 (0.35, 0.76) | 0.76 (0.42, 1.38) | 1.51 (0.97, 2.35) |
| 10+ | 0.62 (0.35, 1.11) | 0.89 (0.34, 2.31) | 1.91 (0.85, 4.29) |

Figure S1: Schoenfeld Residuals for the Matched Cohort. The dashed line and shaded region are a LOESS smooth of the residuals and 95% CI while the dashed black line is the point estimate from the time invariant model and the dotted lines are the 95% CIs for that estimate. Overall, the Schoenfeld residuals did not show concerning deviations from the proportional hazards assumption. We found near zero correlations between time for the Schoenfeld residual for the comparison (TZ/DZ/AZ versus tamsulosin: r = -0.029, p = 0.159; TZ/DZ/AZ versus 5ARI: r = 0.003, p = 0.932; tamsulosin versus 5ARI: r = 0.056, p = 0.023).


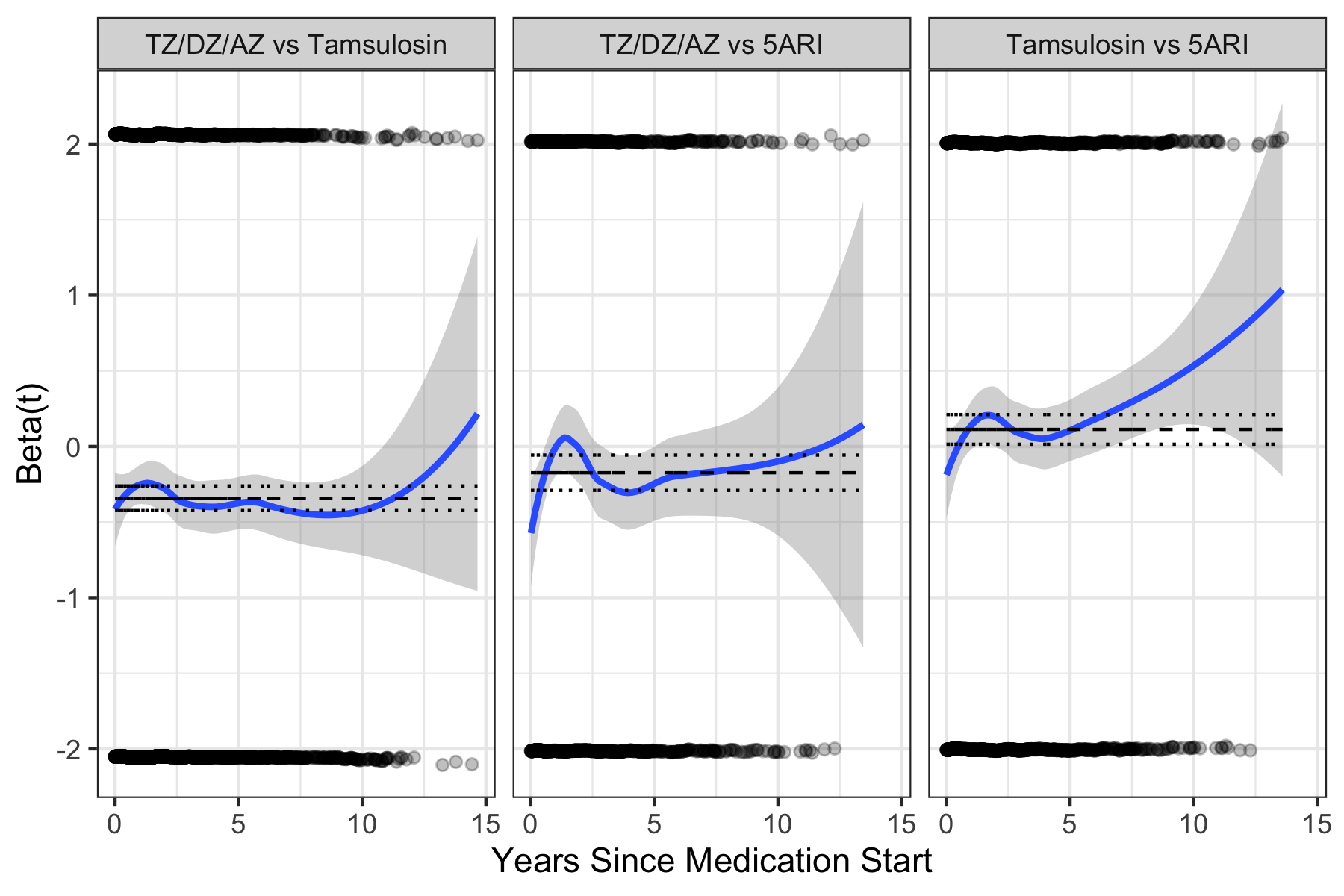


Figure S2: Time Dependent Effects, Schoenfeld Residuals, and Overall HR. The dashed horizonal line is the overall time-invariant estimated hazard ratio and the dotted lines are the 95% CI for that estimated effect. Points are the time-dependent estimates from table S4 with their 95% CI shown as a vertical line. The blue line shows the estimated LOESS fit of the time-dependent estimate based on the Schoenfeld residuals and the shaded gray region is the 95% CI around that fit. Panels are TZ/DZ/AZ vs tamsulosin (A), TZ/DZ/AZ vs 5ARI (B), and tamsulosin vs 5ARI (C). There seems to be no meaningful difference between the non-time-variant and time-variant estimations for TZ/DZ/AZ versus tamsulosin, A. However, it appears the 1-2 year estimate (and neither the 0-1 or 2-3 year estimates) diverge between the estimation procedures for TZ/DZ/AZ vs 5ARI, B. Given the consistency of the estimate on either side, it seems plausible the “bump” is spurious. The tamsulosin vs 5ARI comparison shows estimates much closer to 1.00 during the first few years with a deviation occurring, and increasing, after year 5, C.


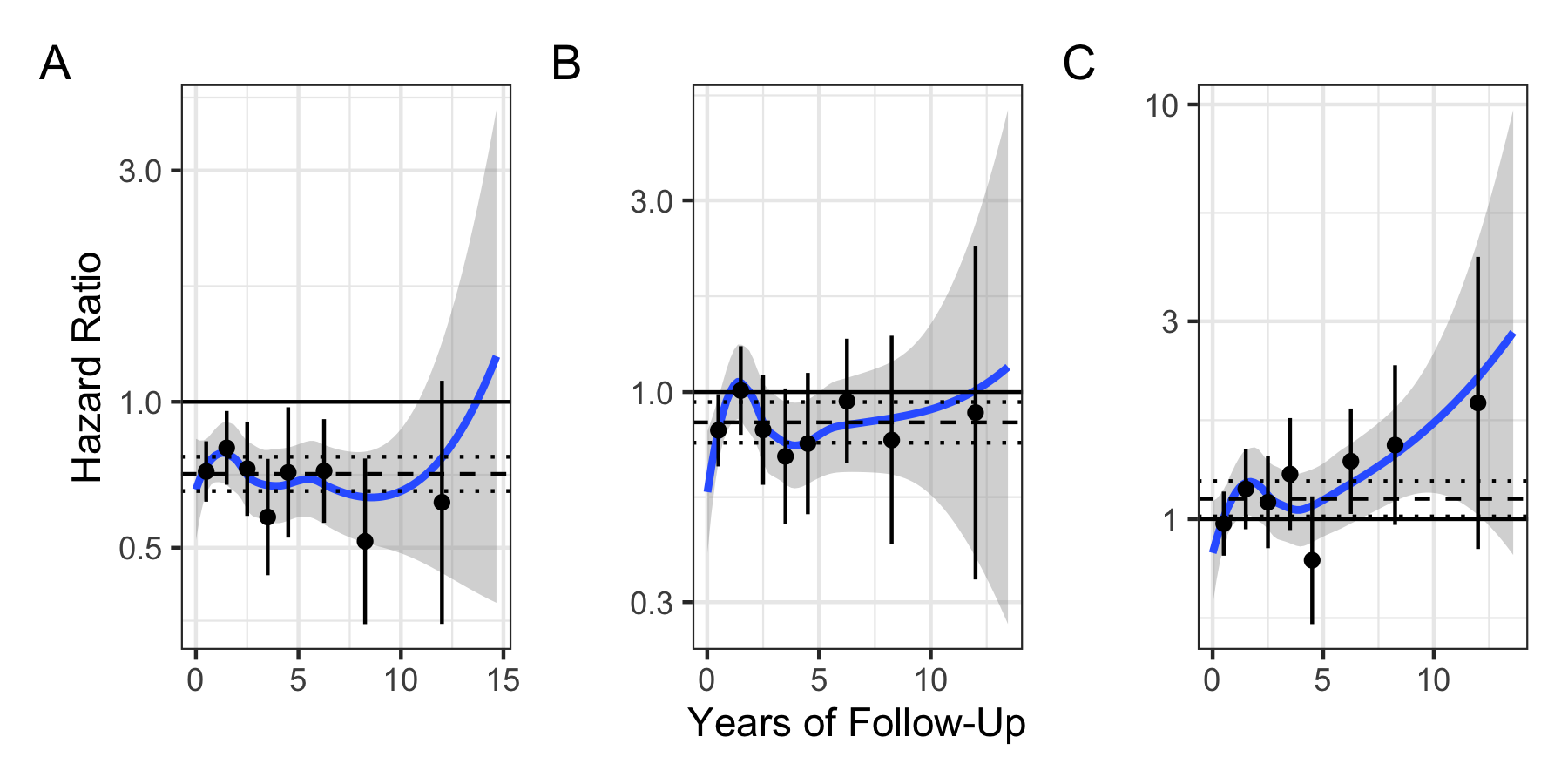


Figure S3: Time-Dependent Log Hazard Ratios by Lead-In Duration. For shorter lead-in durations (0-3 years) there is not consistent evidence of a major departure from the proportional hazards assumption. Longer durations, especially for the TZ/DZ/AZ vs 5ARI comparison, show more deviation but also are very noisy.


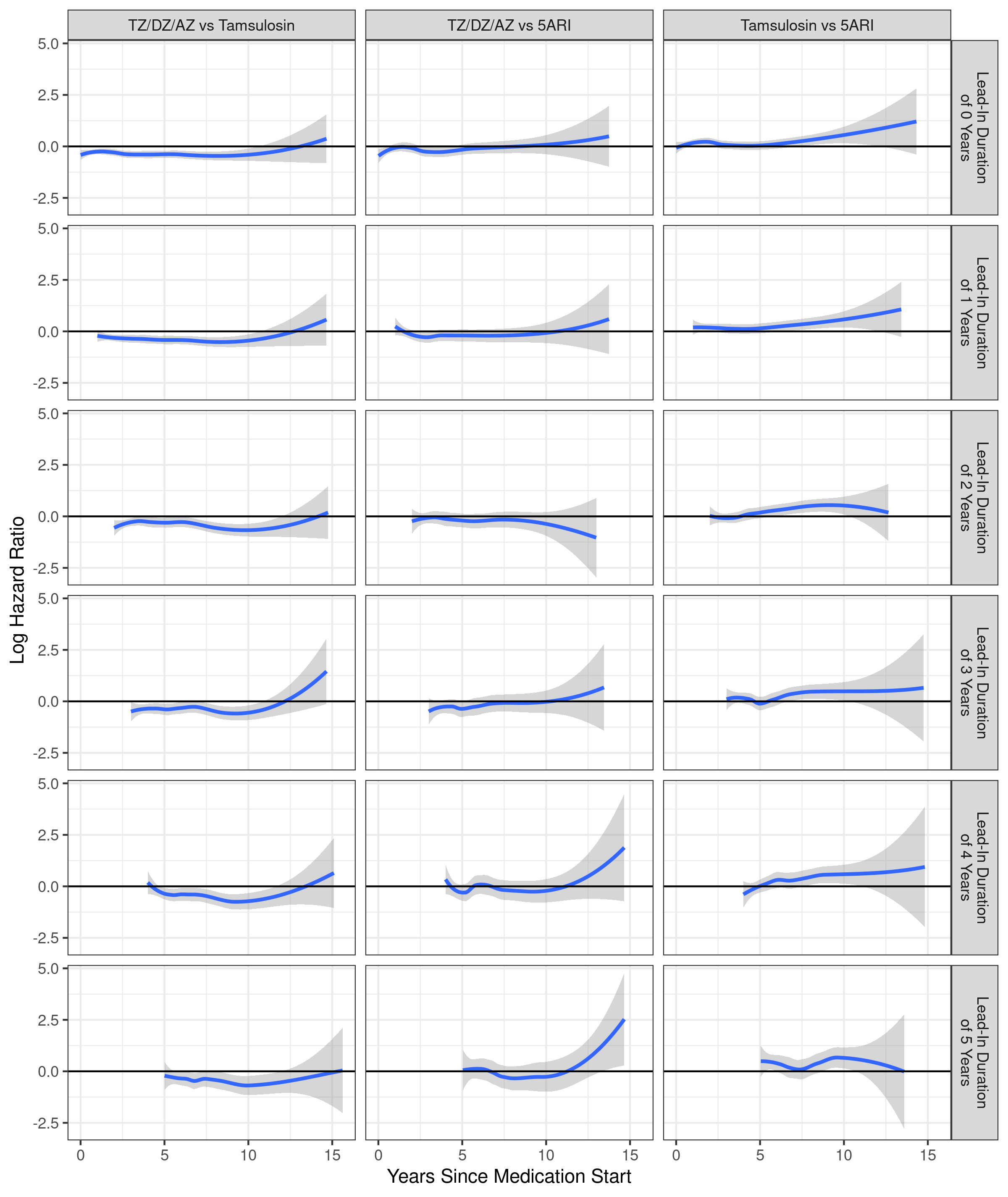


Figure S4: Schoenfeld Residuals at Different Lead-In Windows. In order to assess the deviations shown in Figure S3 for statistical significance, we computed the Pearson’s correlation coefficient between time and the Schoenfeld residuals. We find the correlation is non-significant (95% CI crosses 0) for all the lead-in options for TZ/DZ/AZ versus tamsulosin or 5ARI. The correlation coefficient was statistically significant for several of the tamsulosin vs 5ARI comparison.


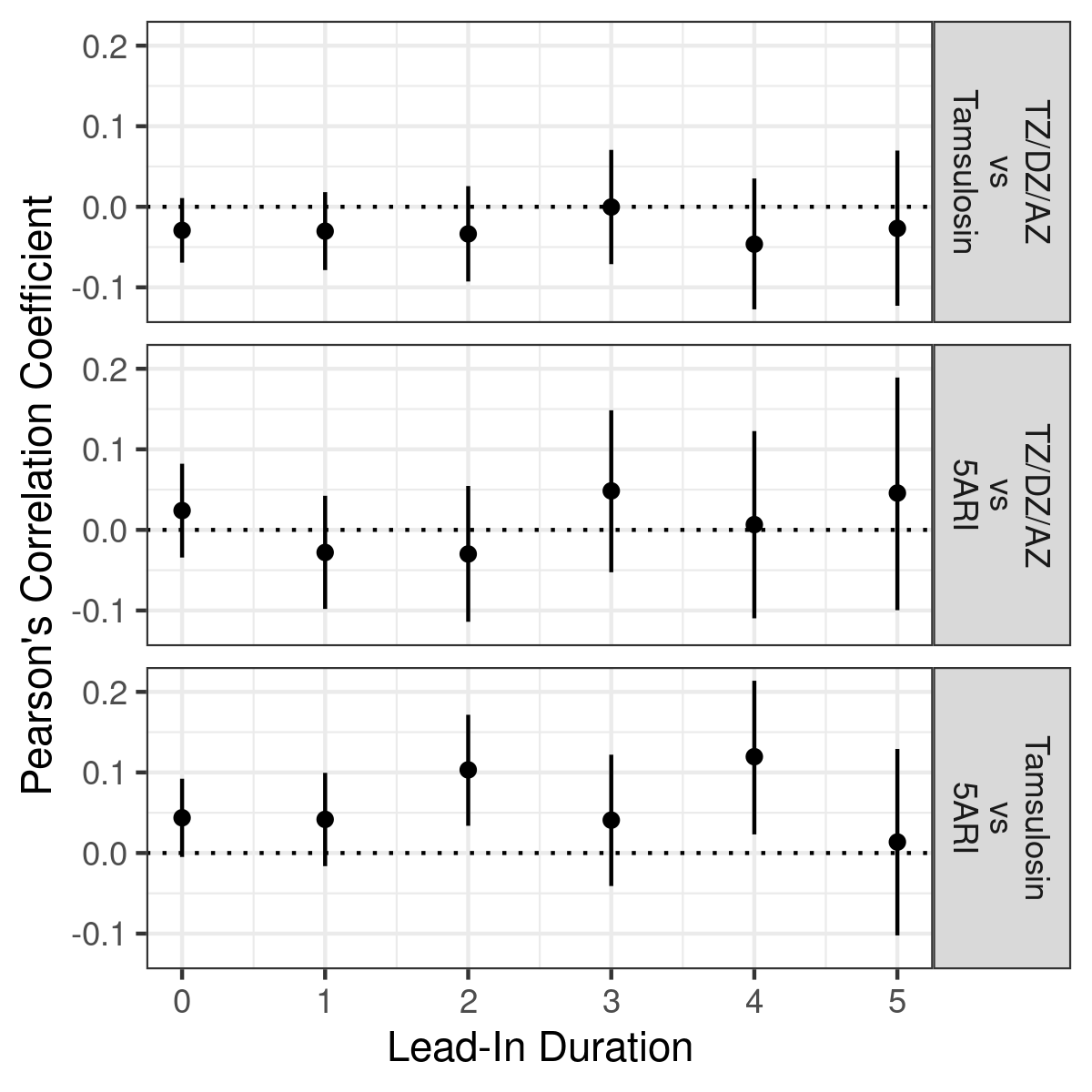

Supplement: Supplementary file 1 — APPENDIX S1. Supporting Information [file MDS-37-2210-s001.docx]
